# High-performing nonlinear visualization of terahertz radiation on a silicon charge-coupled device

**DOI:** 10.1038/ncomms9439

**Published:** 2015-10-26

**Authors:** Mostafa Shalaby, Carlo Vicario, Christoph P. Hauri

**Affiliations:** 1 SwissFEL, Paul Scherrer Institute, 5232 Villigen PSI, Switzerland; 2 École Polytechnique Fédérale de Lausanne, 1015 Lausanne, Switzerland

## Abstract

Photoinduced electron transitions can lead to significant changes of the macroscopic electronic properties in semiconductors. This principle is responsible for the detection of light with charge-coupled devices. Their spectral sensitivity is limited by the semiconductor bandgap which has restricted their visualization capabilities to the optical, ultraviolet, and X-ray regimes. The absence of an imaging device in the low frequency terahertz range has severely hampered the advance of terahertz imaging applications in the past. Here we introduce a high-performing imaging concept to the terahertz range. On the basis of a silicon charge-coupled device we visualize 5–13 THz radiation with photon energy under 2% of the sensor's band-gap energy. The unprecedented small pitch and large number of pixels allow the visualization of complex terahertz radiation patterns in real time and with high spatial detail. This advance will have a great impact on a wide range of terahertz imaging disciplines.

## Abstract (web summary)

The absence of an imaging device in the low frequency terahertz range has hindered the advance of applications. Here, Shalaby *et al*. introduce a silicon based charge coupled device allowing the visualization of terahertz radiation in real time and at high spatial detail.

## Graphical abstract

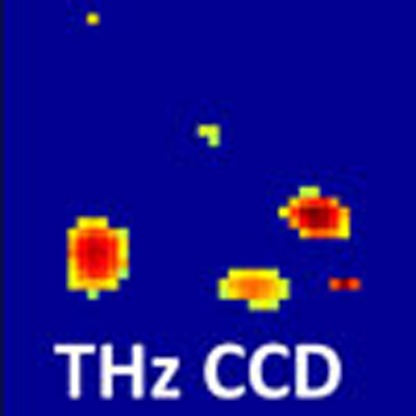

Charge-coupled devices (CCDs) are at the core of many fundamental and advanced imaging methodologies including photography, holography, diffraction imaging, transmission imaging, tomography and fluorescence spectroscopy^1^. CCDs are particularly powerful because they allow for online read-out and single-shot recording at a pixel size of a few micrometers. The typical mode of operation of a CCD sensor depends on an internal photoelectric effect where the incident photons are absorbed in the silicon substrate, generating electron-hole pairs across the bandgap^2^,^3^. A prerequisite for this process is that the incident photon is energetic enough to overcome the bandgap energy *E*_g_. This fundamental limitation on the required photon energy totally prevented the application of CCD sensors to low photon energy/long wavelengths such as terahertz (THz) radiation (0.1–10 THz, *λ*=3–0.03 mm)^4^,^5^,^6^,^7^,^8^.

Given the wide applicability of THz radiation^9^,^10^,^11^,^12^,^13^,^14^,^15^,^16^, imaging in this frequency range is crucial^17^. However, similar to other aspects of the THz technology^18^,^19^,^20^, THz imaging technology and science severely lag behind their counterparts at higher frequency ranges. The efforts to extend the electronic CMOS technology to the THz range is challenged by high complexity, radiation-coupling issues, large pixel size and the limitation to the sub-THz range^4^,^5^,^6^,^7^,^8^. Presently, the state of the art in THz imaging technology relies on a different concept, which is based on a microbolometer array detector with a limited number of pixels (320 × 240) and large pixel size (at best 23.5 μm). The performance is deficient for applications requiring sub-diffraction-limit imaging^21^. Furthermore, the response time is slow, the read-out time is long and the thermal sensor is highly sensitive to fluctuation of the ambient temperature, as the associated thermal energy is in the range of the THz photon energy to be detected (≈25 meV at 290 K, ≈6 THz). In practice, this gives rise to a detector background radiation which can easily vary during exposure time. These limitations call for a more robust and high-performing THz imaging technology.

Here we show that indirect light–matter interaction processes lead to visualization of low-frequency terahertz radiation with photon energy under two per cent of the energy gap. Our work introduces the well-established CCD concept to the THz regime, allowing for THz recording on a large size 1,360 × 1,024 standard silicon chip with a very small pixel size of 4.65 μm. The unprecedented small pitch and large number of pixels allow visualizing complex terahertz radiation patterns in real time and with high spatial detail. While such technology was available in the mid-infrared range (3–15 μm) a corresponding imaging technique in the THz range (15–1,000 μm) has so far been lacking.

## Results

### Experimental technique

Our experiment was performed using a commercially available silicon CCD sensor. Typically, the absorbed photons in the photoactive region (silicon layer) generate electron-hole pairs across the band gap. The electrons generated during the exposure time are confined in a potential well in each pixel before the read-out process is launched. For charge separation to take place, the photon quantum needs to carry sufficient energy to overcome the silicon bandgap energy *E*_g_=1.12 eV, corresponding to a wavelength *λ* of 1.1 μm. In principle, this makes the CCD insensitive to radiation with a wavelength larger than 1.1 μm as the charge-generation region (silicon) becomes transparent. However, some reports extended the use of the CCD sensors into the mid-infrared range, exploiting multi-photon absorption of intense radiation^22^,^23^. As the probability of the latter process drops dramatically for wavelength in the THz range, it is this fundamental limitation which prevented the applications of CCD sensors to long wavelengths. However, the Keldysh theory^24^,^25^,^26^ suggests another process for the creation of free charge carriers based on a tunnelling mechanism when the THz field is strong enough to deform the band structure of the photoactive medium. Although this process is effective only when certain radiation intensity is reached, it is followed by a dramatic increase in free carrier generation efficiency, which finally leads to a very high measured sensitivity.

**Figure 1:**
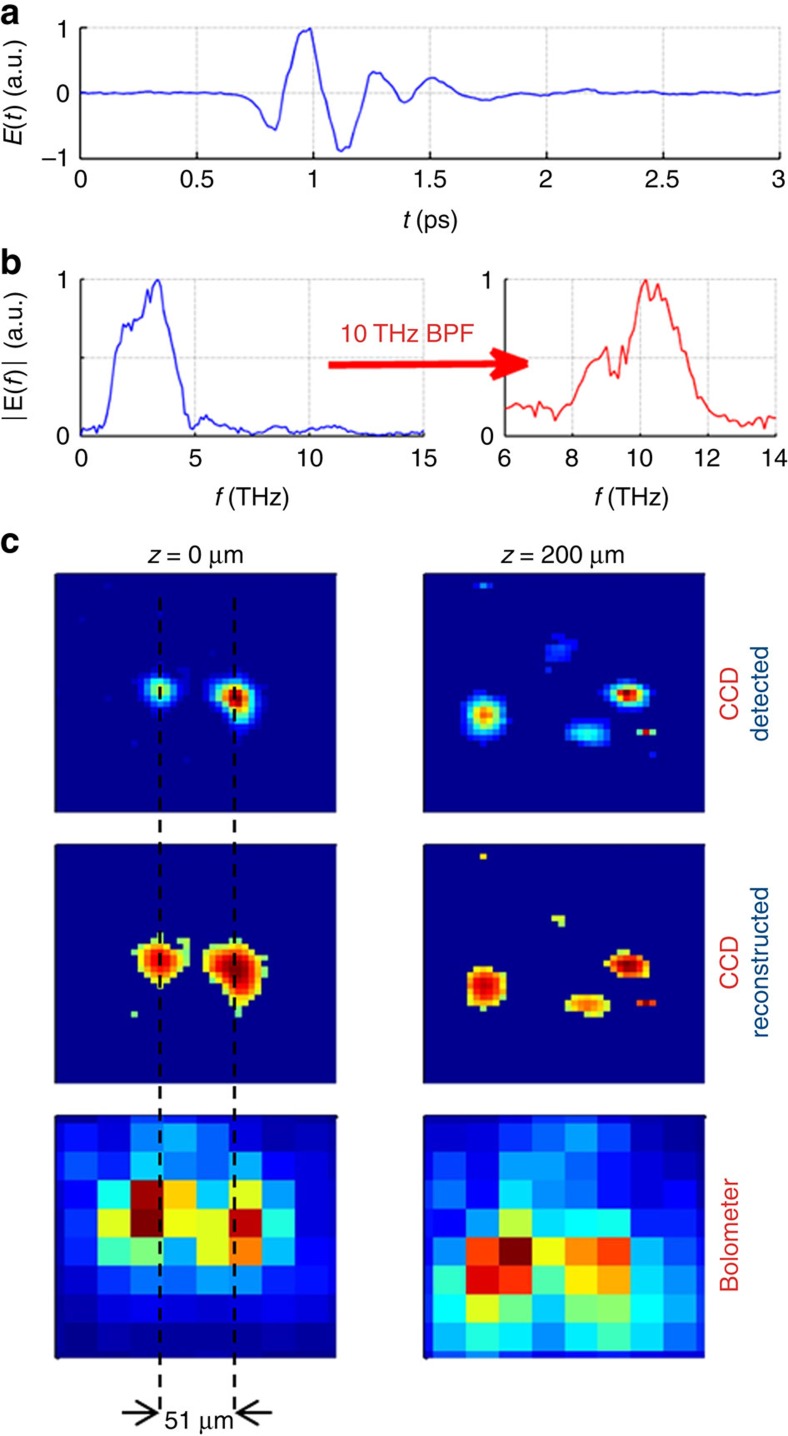
THz characterization with CCD and standard bolometric imager. (**a**) Time-dependent electric field of the THz pulse retrieved using air-biased-coherent-detection technique^27^. (**b**) The corresponding broadband amplitude spectrum. A narrowband portion centered around 10 THz is obtained by application of a band pass filter (BPF). (**c**) The detected images (normalized) on the (4.65-μm pixel size) CCD obtained with the 10 THz BPF in different slices around the focus with *z* being the propagation direction. The corresponding real images reconstructed taking the natural logarithm (as discussed later). The beam consists of several smaller beams that focus in different planes. The images obtained with a (23.5-μm pixel size) bolometric imager are shown. All images are plotted at the same scale on dimension of 200 × 200 μm. In the focal plane (*z*=0 μm), two spots are visible with a separation of 51 μm. While the pixel size is smaller than 51 μm in both cameras and so the two spots were obvious in both images, the high-resolving image from the CCD provides a significantly more detailed and accurate beam profile.

The temporal trace of the exciting THz pulse, generated by optical rectification in a DSTMS organic crystal, is single-cycle (Fig. 1a) and the spectrum is centered around 3 THz with components reaching up to 12 THz (Fig. 1b)^29^,^30^. To eliminate the spectral dependence of our detector, we start our investigations by extracting the spectral components in a narrow bandwidth using a 10 THz-centered band pass filter (BPF) with a full-width at half-maximum bandwidth of 1.45 THz (Fig. 1b). We recorded the image of the focused THz beam on the CCD detector (Fig. 1c) and compare it with the image taken by the state-of-the-art bolometer-based camera (from NEC Inc.).

### Comparison against standard imaging platform

**Figure 2:**
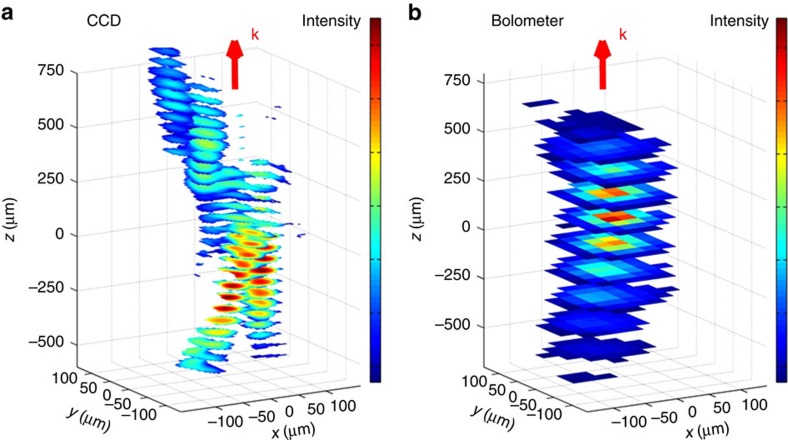
Spatial evolution of a multifaceted THz beam. Images were retrieved across the beam waist using (**a**) CCD and (**b**) bolometric sensors. In the case of CCD, multiple beams with different divergences, sizes and focal planes are clearly visualized. The corresponding images taken from the bolometric detector barely show this detail-rich structure of the beam due to low spatial resolution resulting from the large pixel size. The bolometric sensor has a very strong frequency-dependent sensitivity^28^, but it is still sensitive to the low-frequency components.

As depicted in Figs 1 and 2, the THz beam at the focus consists of several individual beamlets. Although both sensors show this feature, only a diffuse image is observed with the bolometric sensor. In contrast, highly detailed information is provided by the high-resolving CCD sensor. For example, in the assumed focal plane, the two spots are resolved using the CCD to be separated by 51 μm, which corresponds to nearly two pixels from the bolometer array, thus incapable of spatial sampling of the intensity profile. In contrast, the CCD was capable of easily resolving features which are a fraction of the wavelength, down to *λ*/6.5. The shown images are rescaled in intensity using a calibration described below. Furthermore, the CCD sensor offers a signal to noise ratio (SNR) better than 64 for a single-shot exposure and a SNR of 640 for the maximum exposure time of 1 s with the laser system running at 100 Hz. The maximum total energy shone on the detector was 21 μJ. In our experiment, we carried out extensive tests to rule out any contribution from the residual optical pumping beam reaching the detector. This includes the use of different pump filters, non-phase matched pumping of the THz generation crystal, THz attenuation with optically insensitive polarizers, and even the removal of the generation crystal.

### Visualization of complex beam propagation

To get insight on the spatial evolution of the full-THz pulse bandwidth, we show in Fig. 2 the beam profile images recorded with both sensors. Only with the CCD becomes visible the complex propagation behaviour of the multiple beamlets. The beamlets are shown to have different divergences, sizes and focal planes around the assumed focus. In comparison, the large pixel size of the bolometric sensor (Fig. 2b) entirely smears these features out. We mention that no sensor damage was observed in any of the studied configurations.

## Discussion

**Figure 3:**
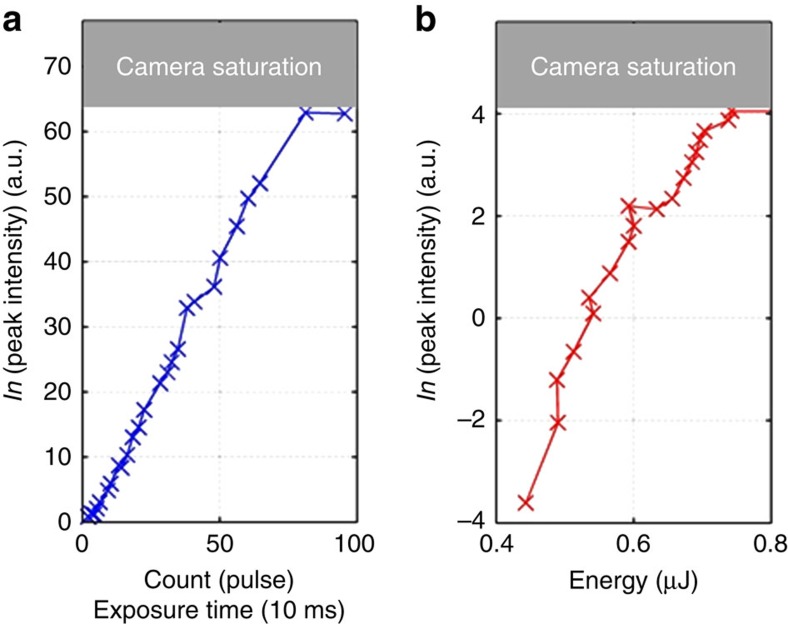
Characterization of the camera response. (**a**) Linear dependence of the camera sensitivity on the exposure time and number of pulses (repetition rate 100 Hz). The exposure time of the image was obtained down to one single shot. (**b**) the dependence of the image intensity on the THz energy using the spectral content obtained from a 10 THz-centered BPF.

We measured the peak intensity of the THz image on the CCD for different exposure times (pulse count) and verified the expected linear response (Fig. 3a). The minimum exposure time was 10 ms, corresponding to a single THz pulse recording at the laser repetition rate of 100 Hz. This linearity verification allows us to extract the dependence of the measured intensity of the CCD on the THz peak intensity/pulse energy. In contrast to the near infrared regime where the response is linear, we obtained a good fit with a (natural) exponential dependence (Fig. 3b for the case of 10 THz BPF). Using the 10 THz BPF, saturation occurs at an energy level ∼700 nJ, focused near the diffraction limit, using the minimum exposure time of 10 ms (single THz pulse).

This sensitivity dependence can be understood by considering the relation between the tunnelling transition rate *ω* and the photon frequency *f*. The tunnelling transition rate to the conduction band for photon energies below the band gap is given^26^ by


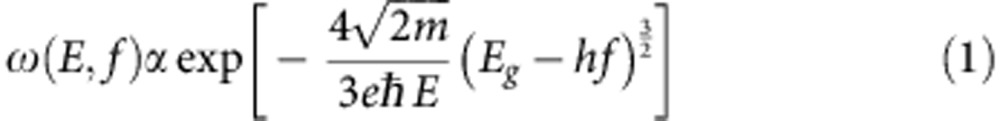


where *E*, *m*, and *h* are the applied electric field, the effective electron mass, and Plank's constant, respectively. Although the above equation indicates nonlinear increase in the released electron-hole pairs with the exciting field, the observed exponential increase in the induced charges (camera sensitivity) is unlikely to be described solely by this mechanism. Another contribution to charge formation could arise from impact ionization^31^, a mechanism that depends on the acceleration of electrons in the substrate impurities under the influence of the high-THz field. When the gained kinetic energy of the carriers reaches a certain threshold, more and more carriers are released due to electron impact. The process can lead to a large increase of carriers with respect to the applied fluence in agreement with our observation^32^. It is likely that both processes contribute sequentially. While tunnelling starts the carrier formation process from the valence and intraband states, impact ionization could occur at a later stage. In Hirori *et al*.^32^, using GaAs quantum wells at low temperature it was found that the number of generated carriers follows the fourth order of THz intensity. Although, at such high nonlinearities, the logarithm at any base of the generated carrier density is likely to fit the THz intensity, we found good agreement with the natural logarithm. Moreover, and similar to our case, saturation of the generated carriers was observed^32^. It was attributed to the enhancement in the Coulomb scattering among the generated carriers. Nevertheless, the band gap of Si and the fact that the sensor is operated at room temperature strongly differentiates the process investigated here from previous studies on THz-induced nonlinearities in semiconductors. Moreover, with the associated THz field, the silicon band gap may slightly change. However, based on previous works, this change is believed to be rather small^26^,^33^. As the exact architecture and design of the commercial CCD was not disclosed by the manufacturer, a conclusive explanation of the underlying physical mechanisms cannot be given.

**Figure 4:**
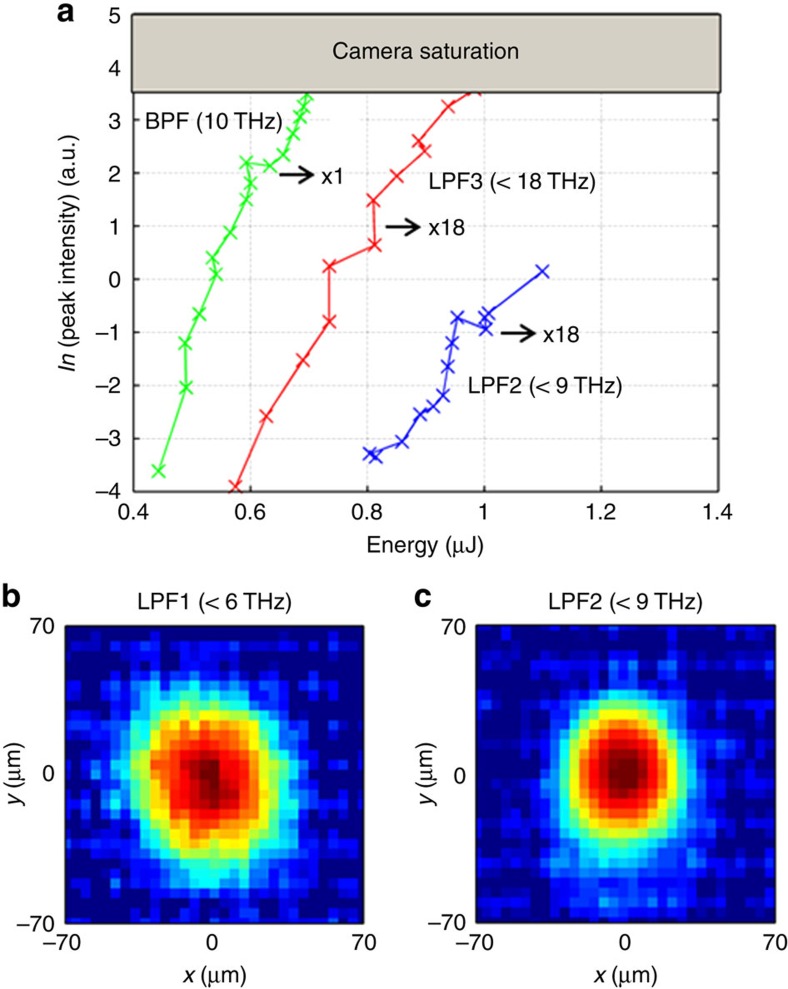
Spectral sensitivity of the CCD camera. (**a**) The dependence of the image intensity on the THz pulse energy in the case of 10-THz band pass filter, BPF (green), 18-THz low pass filter LP3 (red) and 9-THz low pass filter LP2 (blue). The multiplier denotes the required expansion factor of the horizontal axis. The high energy requirements in the latter two cases imply the decrease of sensitivity as the THz frequency decreases. (**b**) Terahertz images reconstructed using CCD in the sub-6 THz and (**c**) sub-9 THz ranges with the unprecedented pixel size of 4.65 μm.

The frequency-dependent sensitivity of the CCD was studied by employing a set of available THz filters including a BPF centered at 10 THz and three low-pass filters with cutoff frequencies at 18 THz (LP3), 9 THz (LP2) and 6 THz (LP1), repectively. As illustrated in Fig. 4a, exponential sensitivity dependence for these frequency regions was found, such as for the BPF (green), the LP3 (red) and for LP2 (blue). From these measurements, it is clear that the CCD shows a frequency-dependent sensitivity which is larger for higher frequency components. Nevertheless the CCD sensitivity at lower frequencies is still sufficient for recording beam profiles at frequencies <9 THz and <6 THz, respectively (Fig. 4b). It is hard to estimate the contributing part of the spectrum below 6 THz to the measured image on the CCD. At the same time, the sensitivity strongly increases with frequency; the power spectral density at 4 THz (Fig. 1b) is around two orders of magnitude higher than that in the range of 5–6 THz.

Finally, it is noteworthy to mention that the presented CCD is not optimized for the THz band and thus requires an enhanced THz flux at that stage. On the basis of the results presented here further optimizations could be envisaged. Coupling the CCD sensor with designed sub-wavelength structures, for example, could lead to very pronounced performance at low THz fluence. Recently reported nanoslit arrays could lead to enhancement by orders of magnitude of the THz radiation. The enhancement factor of these nanoslits features typically a 1/frequency dependence^34^,^35^, which could oppose and even compensate for the frequency-dependent CCD sensitivity shown here.

In conclusion, we have introduced for the first time a high-performing CCD concept to the THz frequency range. Our results overcome the limitations of the presently lagging THz-imaging technology in view of pixel size and detectable frequencies. We have shown that the nonlinear mechanism for the absorption of terahertz radiation could lead to visualization of broadband low-frequency (5–13 THz) radiation by means of a standard silicon CCD detector. The exceptionally small pixel size (4.65 μm) allowed us to unravel the detailed and complicated evolution of a THz multiplet beam profile in space, as a first imaging application. The detector characterization unravels the sensitivity to be exponentially dependent on the THz intensity and to decrease at low frequency. The small pixel size, the single-shot capabilities and the large number of pixels (1.39 Mio) open an avenue for novel THz-imaging applications such as THz holography and tomography, as well as for advanced metrology such as THz wavefront sensors. Many disciplines including fundamental science, medicine and national security will greatly benefit from these advances.

## Methods

For our THz source, we use an optical parametric amplifier system with pulse duration of 65 fs to pump small-size organic crystals DSTMS (thickness of 440 μm and diameter of 6 mm) at 1.5-μm wavelength. The generated THz was focused through an all-reflective telescope assembly based on off axis mirrors with focal distance of 1″, 4″, 2″ and respective diameters of 1″ 2″ 2″. The spectrum of the generated THz pulse is centered around 4 THz. Terahertz pulse detection was performed using Air Biased Coherent Detection. The active CCD sensor of our camera is ICX205AL (Song Inc. Japan).

## Additional information

**How to cite this article:** Shalaby, M. *et al*. High-performing nonlinear visualization of terahertz radiation on a silicon charge coupled device. *Nat. Commun.* 6:8439 doi: 10.1038/ncomms9439 (2015).

## References

[b1] Amelio G. F., Tompsett M. F. & Smith G. E. Experimental verification of the charge coupled device concept. Bell Syst. Tech. J. 49, 593–600 (1970).

[b2] Durini D. High Performance Silicon Imaging: Fundamentals and Applications of CMOS and CCD sensors Elsevier (2014).

[b3] Boyle W. S. & Smith G. E. Charge coupled semiconductor devices. Bell Syst. Tech. J. 49, 587–593 (1970).

[b4] Knap W. Plasma wave detection of sub-terahertz and terahertz radiation by silicon field-effect transistors. Appl. Phys. Lett. 85, 675 (2004).

[b5] Otsuji T., Hanabe M., Nishimura T. & Sano E. A grating-bicoupled plasma-wave photomixer with resonant-cavity enhanced structure. Opt. Express 14, 4815–4825 (2006). 19516639 10.1364/oe.14.004815

[b6] Ojefors E., Pfeiffer U., Lisauskas A. & Roskos H. A 0.65 THz focal-plane array in a quarter-micron CMOS process technology. IEEE J. Solid-St. Circ. 44, 1968–1976 (2009).

[b7] Schuster F. . Broadband terahertz imaging with highly sensitive silicon CMOS detectors. Opt. Express 19, 7827–7832 (2011). 21503093 10.1364/OE.19.007827

[b8] Lee A. W. M. & Hu Q. Real-time, continuous-wave terahertz imaging by use of a microbolometer focal-plane array. Opt. Lett. 30, 2563–2565 (2005). 16208900 10.1364/ol.30.002563

[b9] Kampfrath T., Tanaka K. & Nelson K. A. Resonant and nonresonant control over matter and light by intense terahertz transients. Nat. Photon. 7, 680 (2013).

[b10] Zaks B., Liu R. B. & Sherwin M. S. Experimental observation of electron-hole recollisions. Nature (London) 483, 580 (2012). 22460904 10.1038/nature10864

[b11] Vicario C. . Off-resonant magnetization dynamics phase-locked to an intense phase-stable terahertz transient. Nat. Photon. 7, 720–723 (2013).

[b12] Shalaby M. . Terahertz magnetic modulator based on magnetically clustered nanoparticles. Appl. Phys. Lett. 105, 151108 (2014).

[b13] Cocker T. L. . An ultrafast terahertz scanning tunnelling microscope. Nat. Photon. 7, 620 (2013).

[b14] Liu M. . Terahertz-field-induced insulator-to-metal transition in vanadium dioxide metamaterial. Nature 487, 345 (2012). 22801506 10.1038/nature11231

[b15] Schubert O. . Sub-cycle control of terahertz high-harmonic generation by dynamical Bloch oscillations. Nat. Photon. 8, 119 (2014).

[b16] Cocker T. L. . An ultrafast terahertz scanning tunneling microscope. Nat. Photon. 7, 620–625 (2013).

[b17] Chan W. L., Deibel J. & Mittleman D. M. Imaging with terahertz radiation. Rep. Prog. Phys. 70, 1325–1379 (2007).

[b18] Mochizuki M. & Nagaosa N. Theoretically predicted picosecond optical switching of spin chirality in multiferroics. Phys. Rev. Lett. 105, 147202 (2010). 21230863 10.1103/PhysRevLett.105.147202

[b19] Shalaby M. . Terahertz macrospin dynamics in insulating ferrimagnets. Phys. Rev. B 88, 140301 (R) (2013).

[b20] Akyildiz I. F., Jornet J. M. & Han C. Terahertz band: next frontier for wireless communications. Phys. Commun. J. 12, 16–32 (2014).

[b21] Moon K. . Subsurface nanoimaging by broadband terahertz pulse near-field microscopy. Nano Lett. 15, 549 (2015). 25436437 10.1021/nl503998v

[b22] Zavriyev A., Dupont E., Corkum P. B., Liu H. C. & Biglov Z. Imaging and autocorrelation of ultrafast infrared laser pulses in the 3–11-μm range with silicon CCD cameras and photodiodes. Opt. Lett. 20, 1886–1888 (1995).19862191

[b23] Briggman K. A., Richter L. J. & Stephenson J. C. Imaging and autocorrelation of ultrafast infrared laser pulses in the 3–11-μm range with silicon CCD cameras and photodiodes. Opt. Lett. 26, 238–240 (2001). 18033559 10.1364/ol.26.000238

[b24] Keldysh L. V. Ionization in the field of a strong electromagnetic wave. J. Exp. Theor. Phys. 47, 1945 (1964).

[b25] Lange C. . Extremely nonperturbative nonlinearities in gaas driven by atomically strong terahertz fields in gold metamaterials. Phys. Rev. Lett. 113, 227401 (2014). 25494089 10.1103/PhysRevLett.113.227401

[b26] Goodfellow J. . Below gap optical absorption in GaAs driven by intense, single-cycle coherent transition radiation. Opt. Express 22, 17423–17429 (2014). 25090555 10.1364/OE.22.017423

[b27] Dai J., Xie X. & Zhang X.-C. Detection of broadband terahertz waves with a laser-induced plasma in gases. Phys. Rev. Lett. 97, 103903 (2006). 17025819 10.1103/PhysRevLett.97.103903

[b28] Naoki O. . in *Microbolometer Terahertz Focal Plane Array and Camera with Improved Sensitivity at 0.5-0.6 THz* (*39th International Conference on Infrared, Millimeter, and Terahertz waves (IRMMW-THz)*) (Tucson, AZ, USA, 2014).

[b29] Shalaby M. & Hauri C. P. Demonstration of a low frequency three-dimensional terahertz bullet with extreme brightness. Nat. Commun. 6, 5976 (2015). 25591665 10.1038/ncomms6976

[b30] Vicario C., Monoszlai B. & Hauri C. P. GV/m single-cycle terahertz fields from a laser-driven large-size partitioned organic crystal. Phys. Rev. Lett. 112, 213901 (2014).

[b31] Hoffmann M. C., Hebling J., Hwang H. Y., Yeh K.–L. & Nelson K. A. Impact ionization in InSb probed by terahertz pump—terahertz probe spectroscopy. Phys. Rev. B 79, 161201 (R) (2009).

[b32] Hirori H. . Extraordinary carrier multiplication gated by a picosecond electric field pulse. Nat. Commun. 2, 594 (2011). 22186890 10.1038/ncomms1598 PMC3247824

[b33] Ghimire S. . Redshift in the optical absorption of ZnO single crystals in the presence of an intense midinfrared laser field. Phys. Rev. Lett. 107, 167407 (2011). 22107430 10.1103/PhysRevLett.107.167407

[b34] Shalaby M. . Skirting terahertz waves in a photo-excited nanoslit structure. Appl. Phys. Lett. 104, 171115 (2014).

[b35] Seo M. . Terahertz field enhancement by a metallic nano slit operating beyond the skin-depth limit. Nat. Photon. 3, 152–156 (2009).

